# Dynamic Responses of Electrically Driven Quartz Tuning Fork and qPlus Sensor: A Comprehensive Electromechanical Model for Quartz Tuning Fork

**DOI:** 10.3390/s19122686

**Published:** 2019-06-14

**Authors:** Manhee Lee, Bongsu Kim, Sangmin An, Wonho Jhe

**Affiliations:** 1 Department of Physics, Chungbuk National University, Cheongju, Chungbuk 28644, Korea; 2 Department of Physics and Astronomy, Seoul National University, Gwanak-gu, Seoul 08826, Korea; rolliney@hanmail.net (B.K.); jmk8755@snu.ac.kr (S.A.)

**Keywords:** quartz tuning fork, qPlus, atomic force microscopy, sensor, 77.65.Fs, 68.37.Ps, 42.79.Pw, 05.45.-a

## Abstract

A quartz tuning fork and its qPlus configuration show different characteristics in their dynamic features, including peak amplitude, resonance frequency, and quality factor. Here, we present an electromechanical model that comprehensively describes the dynamic responses of an electrically driven tuning fork and its qPlus configuration. Based on the model, we theoretically derive and experimentally validate how the peak amplitude, resonance frequency, quality factor, and normalized capacitance are changed when transforming a tuning fork to its qPlus configuration. Furthermore, we introduce two experimentally measurable parameters that are intrinsic for a given tuning fork and not changed by the qPlus configuration. The present model and analysis allow quantitative prediction of the dynamic characteristics in tuning fork and qPlus, and thus could be useful to optimize the sensors’ performance.

## 1. Introduction

Understanding the dynamics of a probe’s motion is important in order to use the probe as a quantitative force sensor in atomic force microscopy and spectroscopy [1,2]. For probes such as micro-cantilevers and quartz tuning forks (TFs), there have been long-investigated linear and nonlinear dynamics [3,4,5] and associated models [6] of the probes. Various mechanistic models of micro-cantilevers have been suggested based on a simple harmonic oscillator [7], a multi-mode oscillator [8], or normal and torsional deflections of a three-dimensional beam [9]. For TFs, the qPlus configuration [10] is well-approximated as the harmonic oscillator when it is driven mechanically [11,12] so that the qPlus sensor facilitates quantitative force measurement, and thus it is widely employed for dynamic force spectroscopy [13].

While the mechanically driven qPlus allows analytical description of the probe dynamics, one could use the electrically driven qPlus (ED-qPlus) sensor to exploit the capability of self-actuation and self-detection. The traditional equivalent circuit model for piezoelectric resonators [14] can be used to describe the motion of both the ED-qPlus and electrically driven TFs (ED-TFs). However, the dynamic characteristics of the ED-qPlus such as peak amplitude, resonance frequency, and quality factor are very distinct from those of the original form (i.e., bare TF), although they are both electrically driven and only one of the two prongs is fixed in the qPlus. Obviously, the one fixed prong affects the overall electromechanical characteristics in the qPlus configuration, but it is not quantitatively understood how the characteristics alter by transforming TF to qPlus.

In this paper, we present an electromechanical model that comprehensively describes the motions of the ED-TF and ED-qPlus. Our model predicts the changes in peak amplitude, resonance frequency, quality factor, and normalized capacitance from the TF to its qPlus configuration, and we confirm the changes experimentally. Furthermore, we introduce two intrinsic constants that are independent of the probe type, TF or qPlus. Our results could be useful to optimize sensors’ dynamic characteristics for quantitative interaction measurements with qPlus or TF.

## 2. Experiment

**Figure 1:**
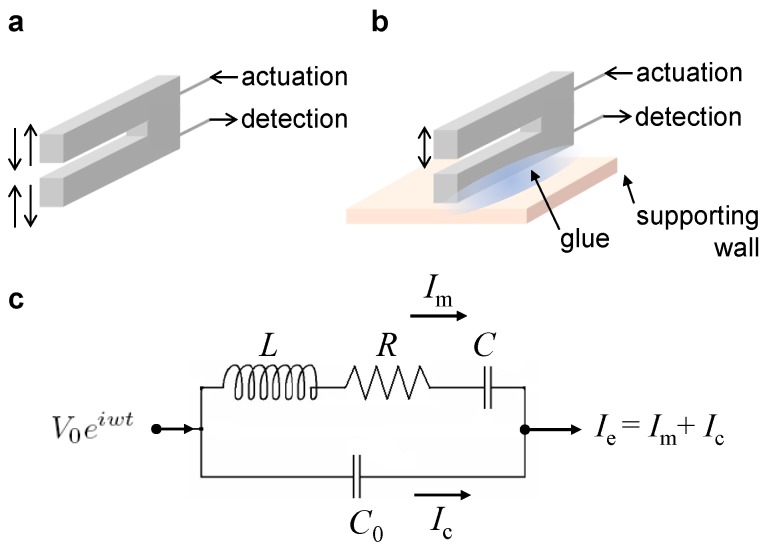
Two working configurations of electrically driven quartz tuning forks and the traditional electrical circuit model for quartz resonators. (**a**) The quartz tuning fork with two prongs can be electrically actuated and its dynamic response can also be measured electrically, where the two prongs move in opposite directions, in an antisymmetric mode of vibration; (**b**) The qPlus sensor is made by fixing one prong firmly to a supporting wall so that the other prong is allowed to vibrate. Both the actuation and the detection of the qPlus can also be made electrically; (**c**) The equivalent circuit model for quartz resonators (e.g., tuning fork, qPlus).

A quartz tuning fork is an electromechanical resonator, originally developed for the clock generators of quartz watches and now widely used as force sensors in atomic force microscopy (AFM) [15,16], near-field scanning optical microscopy [17], electrostatic force microscopy [18], and magnetic force microscopy [19]. The TF can be excited electrically, and its mechanical vibration can also be measured electrically, as described in Figure 1a. Due to the capability of self-actuation and self-detection, neither an external actuator to drive the TF nor an optical setup to detect its motion are required. When using a TF as a quantitative force sensor, one should carefully analyze the response signal of the TF, because the two prongs are coupled with each other. One way to eliminate the coupling between the two prongs is to firmly fix one prong to a supporting wall. This TF configuration is called qPlus [10], as schematically described in Figure 1b.

The electrical responses of the ED-TF (Figure 1a) and ED-qPlus (Figure 1b) are described well by the equivalent circuit model shown in Figure 1c. Note that the equivalent circuit describes the linear motion of a TF or qPlus, although a recent study showed the amplitude dependence of the resonance frequency in such quartz resonators [20]. Therefore, one should consider the nonlinearity when using a quartz sensor to quantify the tip–sample interaction potentials and forces with milli-electron volt and pico-Newton resolutions [20]. In the equivalent circuit, the LRC circuit is connected in parallel with a capacitance $C_{0}$, and thus the total signal $I_{e}$ is given by the sum of $I_{m}$ and $I_{c}$. While the current through the LRC circuit $I_{m}$ represents the vibrational motion of the TF or qPlus, the $C_{0}$ produces the stray capacitance current $I_{c}$. Therefore, the total signal $I_{e}$ includes both the motion-induced signal $I_{m}$ and the capacitive signal $I_{c}$. Based on the model (Figure 1c), we can derive the resonance curve function, the oscillation amplitude $A_{e}$ versus the driving frequency f(=w/2π) [3], for the measured electrical signal $I_{e}$:

(1) $$\begin{array}{cc} A_{e}= & \mathrm{Abs}\left[\left(\frac{1}{1-{\left(\frac{w}{w_{0}}\right)}^{2}+i\left(\frac{w}{w_{0}Q}\right)}+{\bar{C}}_{0}\right)\frac{A_{0}w}{Qw_{0}}\right] \end{array}$$

(2) $$\begin{array}{cc} = & \frac{A_{0}w}{Qw_{0}}\sqrt{\frac{1+2{\bar{C}}_{0}\left(1-\frac{w^{2}}{w_{0}^{2}}\right)+{\left({\bar{C}}_{0}\right)}^{2}{\left(1-\frac{w^{2}}{w_{0}^{2}}\right)}^{2}+{\left({\bar{C}}_{0}\right)}^{2}{\left(\frac{w}{w_{0}Q}\right)}^{2}}{{\left(1-\frac{w^{2}}{w_{0}^{2}}\right)}^{2}+{\left(\frac{w}{w_{0}Q}\right)}^{2}}}, \end{array}$$

where $A_{0}=R_{\mathrm{out}}\left(V_{0}/R\right)$, $w_{0}=1/\sqrt{LC}$, $Q=Lw_{0}/R$, and ${\bar{C}}_{0}=C_{0}/C$. Here, $R_{\mathrm{out}}$ is the resistance that regulates the magnitude of output voltage, given by the combination of the preamplifier gain and the controlled gain of the voltage divider at the input of the measuring instrument.

**Figure 2:**
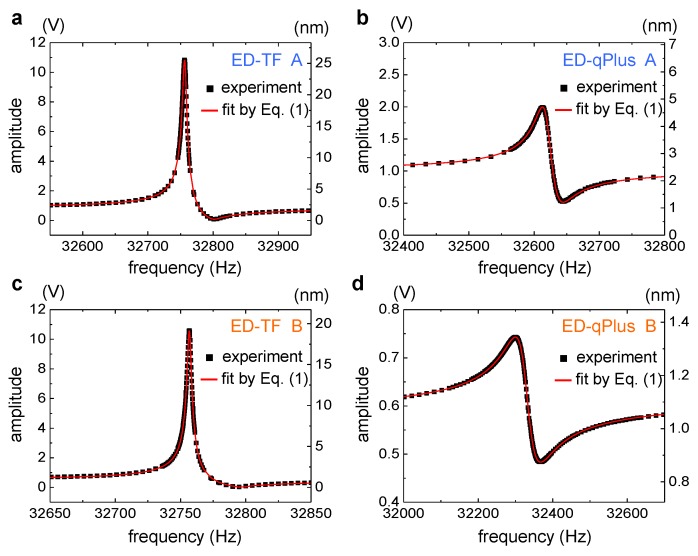
Electrically measured resonance curves of the bare tuning fork and its qPlus configuration (both electrically driven). The electrical signals of the tuning fork (**a**,**c**) and qPlus (**b**,**d**) showed asymmetric curves with one peak and one local minimum. These curves were well fit by Equation (1) derived from the equivalent circuit model (Figure 1c). From each fit, we could uniquely determine the dynamic characteristics shown in Equation (1), $A_{0},Q,f_{0}\left(=w_{0}/2\pi \right)$, and ${\bar{C}}_{0}\left(=C_{0}/C\right)$, for each probe. We used two tuning forks with different sizes, electrically driven tuning fork (ED-TF) A and ED-TF B (**a**,**c**), and they were transformed to qPlus sensors, ED-qPlus A and ED-qPlus B (**b**,**d**), respectively. The electrical signal in volts (left vertical axis) was converted into mechanical amplitude in nanometers (right vertical axis) by using a theoretical method (method (a) described in Ref. [16]). For accurate calibration of oscillation amplitude, one can use experimental methods based on thermal noise spectrum (method (b) in Ref. [16]) for qPlus sensors and energy balance principle [21] generally for quartz resonators, rather than the theoretical approach.

Figure 2 shows the experimentally measured resonance curves of the ED-TF and ED-qPlus, where we used two TFs of different size, TF A (Figure 2a) and TF B (Figure 2c), and their qPlus configurations, qPlus A (Figure 2b) and qPlus B (Figure 2d), respectively. All probes were electrically driven and their motion was also electrically detected. The resonance curve exhibited one peak and one local minimum for all probes. The peak originated from the vibrational resonance of the mechanical motion of the probe $I_{m}$, and the local minimum from the stray capacitance current $I_{c}$. With one prong fixed, the resonance curve of the qPlus showed significant change in the overall shape; the peak amplitude of the ED-qPlus decreased by an order of magnitude, and the bandwidth and the resonance frequency decreased compared to the ED-TF (Figure 2).

**Figure 3:**
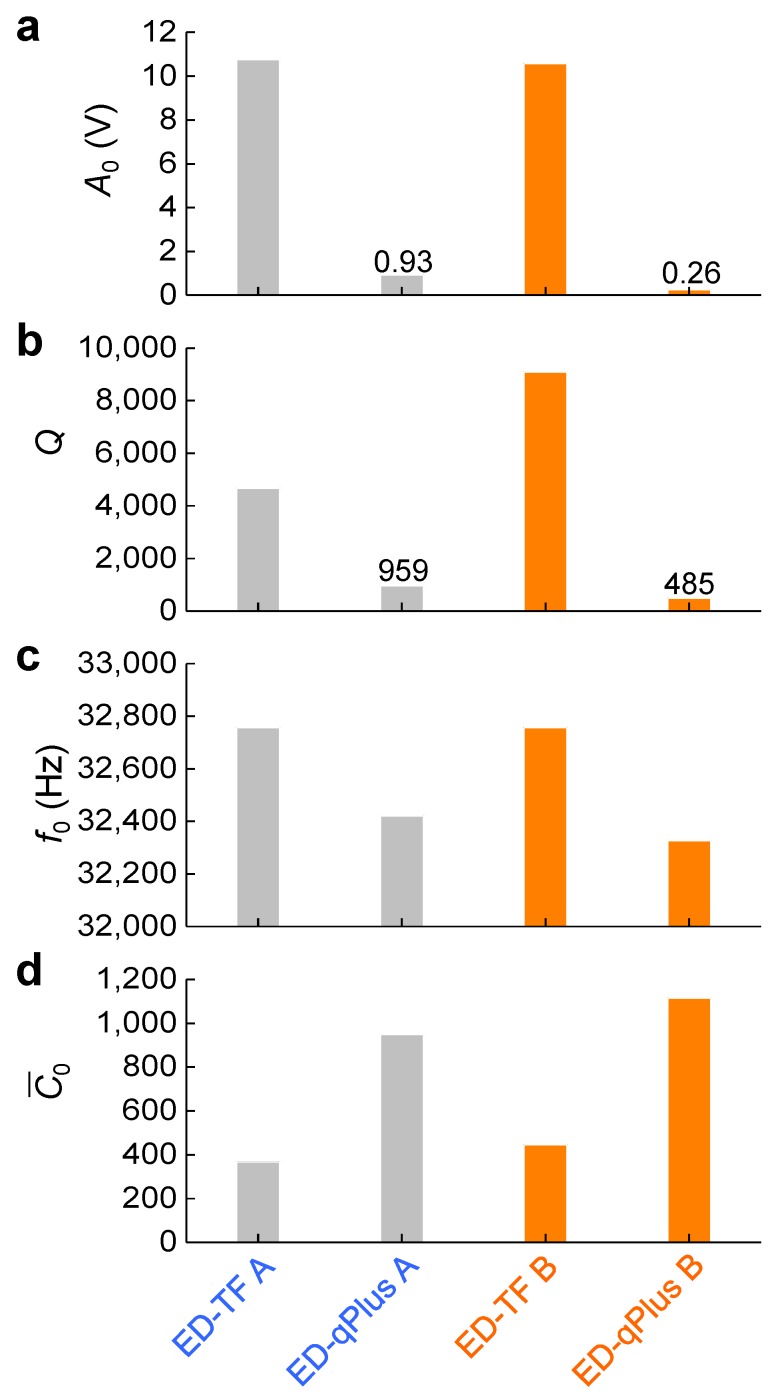
Dynamic characteristics of the electrically driven tuning fork and qPlus sensor. From the resonance curves (Figure 2) and their fit curves using Equation (1), one can uniquely determine the four dynamic characteristics, (**a**) $A_{0}$, (**b**) *Q*, (**c**) $f_{0}$, and (**d**) ${\bar{C}}_{0}$.

Although only one prong is fixed in the qPlus, the dynamic characteristic parameters of the qPlus were very different from that of the TF, as shown in Figure 3. We could uniquely determine the dynamic characteristics, $A_{0},Q,f_{0}\left(=w_{0}/2\pi \right)$, and ${\bar{C}}_{0}$, by fitting Equation (1) to the experimentally measured resonance curves shown in Figure 2. For probes A and B, we found that the qPlus configuration showed much lower values of peak amplitude $A_{0}$ (Figure 3a), quality factor *Q* (Figure 3b), and resonance frequency $f_{0}$ (Figure 3c) than the original bare TF. On the other hand, the capacitance ${\bar{C}}_{0}$ (Figure 3d) was about twice that of the TF for both probes A and B. As we will show, this was not accidental but reflects the geometrical structures and mechanics of the qPlus and the TF.

The equivalent circuit model provides the electrical parameters L,R,C, and $C_{0}$ that reproduce the dynamic characteristics of the ED-TF and the ED-qPlus such as $A_{0},Q,f_{0}\left(=w_{0}/2\pi \right)$, and ${\bar{C}}_{0}$, but it does not give information about how the electrical parameters are quantitatively related to the mechanical parameters of the TF or qPlus (e.g., the prong’s stiffness). Thus, we cannot explain how the electrical parameters and the electrical responses are changed by the qPlus configuration solely based on the electrical model. Moreover, the equivalent circuit model does not include detailed information about the mechanical motion of the TF or qPlus.

## 3. Theory and Analysis

**Figure 4:**
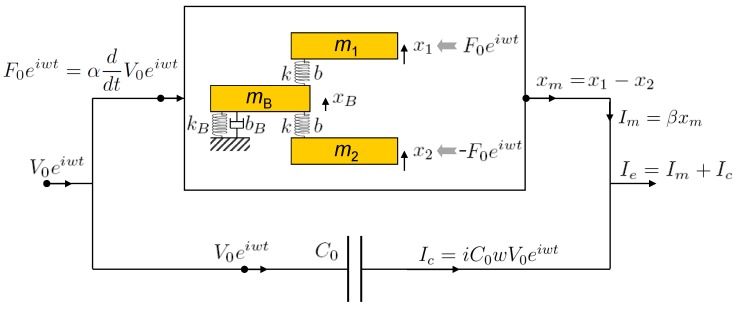
Electromechanical model for an electrically driven tuning fork. The input voltage $V_{0}e^{iwt}$ effectively generates the mechanical force $F_{0}e^{iwt}$, given as $i\alpha wV_{0}e^{iwt}$, where α is the constant that converts applied electrical voltage to mechanical force. The force actuates the two prongs of the tuning fork, and the geometrical displacement of the two prongs $x_{m}=x_{1}-x_{2}$ induces the electrical current $I_{m}=\beta x_{m}$, where β is the converting factor. In addition, the input voltage induces the stray capacitance current $I_{c}$. The measured current $I_{e}$ is given by the sum of the motion-induced current and the stray capacitance current, that is, $I_{e}=I_{m}+I_{c}$ (see the text for details).

We present a comprehensive electromechanical model of a tuning fork, as described in Figure 4. Several mechanical models have been reported for the tuning fork and qPlus. Since Naber et al. presented a mechanical model including two masses and three springs [22], various models with the base damping [23] and the base mass [24] were proposed. While several models employ a spring to directly couple two prongs [25], our model shows the coupling of prongs by the common link to the base, as shown in Figure 4.

The qPlus configuration of the TF is obtained by fixing one prong, here the lower prong in Figure 4, which is experimentally obtained by attaching the prong to a supporting wall (Figure 1b) and here theoretically obtained by increasing the mass $m_{2}\to \infty$ (see Appendix A for another possible way to obtain the qPlus configuration). Therefore, the model in Figure 4 is generally for both the ED-TF and ED-qPlus. When applying a voltage $V_{0}e^{iwt}$ to the probe, the voltage signal effectively exerts a force $F_{0}e^{iwt}$ on the two prongs of the TF. Then, the two prongs with masses $m_{1}$ and $m_{2}$ and the base with $m_{B}$ vibrate, and the mechanical motion of the two prongs induces a current $I_{m}$. At the same time, the stray capacitance current given by $I_{c}=iC_{0}wV_{0}e^{iwt}$ also flows, which is associated with the electrical structure of the probes and the applied voltage $V_{0}e^{iwt}$. Thus, the total electrical signal $I_{e}$ is given by the sum of $I_{m}$ and $I_{c}$.

The model (Figure 4) employs two conversion factors, α and β, which can be obtained by experimental calibration of the probe system [26]. The factor α is the constant that converts driving voltage $V_{0}e^{iwt}$ to mechanical force $F_{0}e^{iwt}$ applied to the TF or qPlus, such as $F_{0}e^{iwt}=\alpha V_{0}iwe^{iwt}$. The β is the constant converting geometrical displacements of the two prongs, $x_{m}=x_{1}-x_{2}$, to electrical current $I_{m}$ (i.e., $I_{m}=\beta x_{m}$).

By solving the coupled motion of two prongs and the base (Figure 4), we obtain $x_{m}\;\left(=x_{1}-x_{2}\right)$ and thus the experimentally measurable signal $I_{m}$ or $I_{e}$. If the masses of the two prongs are same, $m_{1}\approx m_{2}$, then the motion of the two prongs is expected to be antisymmetric, $x_{1}\approx -x_{2}$, and the base motion negligible $x_{B}\approx 0$. The antisymmetric motion of the two prongs induces an electrical signal $I_{m}=2\beta x_{1}$, and thus the magnitude of the measured voltage signal, $I_{e}R_{\mathrm{out}}\left(=\left(I_{m}+I_{c}\right)R_{\mathrm{out}}\right)$, is simply given as:

(3) $$\begin{array}{c} A_{e}^{\mathrm{TF}}=\mathrm{Abs}\left[\left(\frac{1}{1-{\left(\frac{w}{w_{0}^{\mathrm{TF}}}\right)}^{2}+i\left(\frac{w}{w_{0}^{\mathrm{TF}}Q^{\mathrm{TF}}}\right)}+\frac{C_{0}k}{2\alpha \beta }\right)\frac{2\alpha \beta wV_{0}R_{\mathrm{out}}}{k}\right]. \end{array}$$

Here, the motion of the two prongs is antisymmetric, so $w_{0}^{\mathrm{TF}}=w_{0}=\sqrt{k/m}$ and $Q^{\mathrm{TF}}=k/\left(bw_{0}\right)$.

On the other hand, the qPlus sensor allows only one prong to vibrate, here $x_{1}$, and the motion of the prong $x_{1}$ is highly damped by the interaction with the base (Figure 4). Although the exact motion of the prong $x_{1}$ can be solved analytically from the model in Figure 4, we notice that the resulting motion of the qPlus would be approximated as a harmonic motion with altered resonance frequency $w_{0}^{\mathrm{qPlus}}$ and quality factor $Q^{\mathrm{qPlus}}$. By solving the coupled equations of motion for $x_{1}$ and $x_{B}$, we obtain the current $I_{e}=\beta x_{1}+I_{c}$ of the qPlus, and the magnitude of measured voltage $R_{\mathrm{out}}I_{e}$ is then given as follows:

(4) $$\begin{array}{cc} A_{e}^{\mathrm{qPlus}}= & \mathrm{Abs}\left[\left(\frac{1}{1-{\left(\frac{w}{w_{0}^{\mathrm{qPlus}}}\right)}^{2}+i\left(\frac{w}{w_{0}^{\mathrm{qPlus}}Q^{\mathrm{qPlus}}}\right)}+\frac{C_{0}k}{\alpha \beta }\right)\frac{\alpha \beta wV_{0}R_{\mathrm{out}}}{k}\right], \end{array}$$

with the resonance frequency $w_{0}^{\mathrm{qPlus}}$ and quality factor $Q^{\mathrm{qPlus}}$ (please see Appendix A for details):

(5) $$\begin{array}{cc} \frac{w_{0}^{\mathrm{qPlus}}}{w_{0}^{\mathrm{TF}}} & \approx \sqrt{\frac{k_{B}/k+1}{k_{B}/k+2}}, \end{array}$$

(6) $$\begin{array}{cc} \frac{Q^{\mathrm{qPlus}}}{Q^{\mathrm{TF}}} & \approx \frac{k_{B}/k}{k_{B}/k+H\;}, \end{array}$$

where *H* is a constant related with the damping ratio $b_{B}/b$, on the order of $10^{2}$ (please see Appendix A for details).

Equations (5) and (6) predict both the resonance frequency and the quality factor of the qPlus sensor decrease, compared to that of the original TF, depending on the ratio of base stiffness to prong stiffness ($k_{B}/k$) and the value of *H*. The reduction of the resonance frequency and quality factor were consistently observed in our experiment (Figure 3).

In Equation (3), the first term represents the mechanical motion of the prongs of the TF, whereas the second term indicates the stray capacitance current. Similarly, the mechanical motion of the qPlus is shown in the first term of Equation (4) and the capacitance in the second term of Equation (4). Therefore, the mechanical peak amplitude $A_{0}^{\mathrm{TF}}$ for the TF and $A_{m}^{\mathrm{qPlus}}$ for the qPlus are:

(7) $$\begin{array}{cc} & A_{m}^{\mathrm{TF}}=\frac{2\alpha \beta w_{0}^{\mathrm{TF}}Q^{\mathrm{TF}}V_{0}R_{\mathrm{out}}}{k}, \end{array}$$

(8) $$\begin{array}{cc} & A_{m}^{\mathrm{qPlus}}=\frac{\alpha \beta w_{0}^{\mathrm{qPlus}}Q^{\mathrm{qPlus}}V_{0}R_{\mathrm{out}}}{k}, \end{array}$$

and the normalized capacitance, ${\bar{C}}_{0}^{\mathrm{TF}}$ and ${\bar{C}}_{0}^{\mathrm{qPlus}}$:

(9) $$\begin{array}{cc} & {\bar{C}}_{0}^{\mathrm{TF}}=\frac{C_{0}k}{2\alpha \beta }, \end{array}$$

(10) $$\begin{array}{cc} & {\bar{C}}_{0}^{\mathrm{qPlus}}=\frac{C_{0}k}{\alpha \beta }. \end{array}$$

Since $w_{0}^{\mathrm{TF}}>w_{0}^{\mathrm{qPlus}}$ (Equation (5)), the peak amplitude of the TF $A_{0}^{\mathrm{TF}}$ is higher than that of qPlus $A_{0}^{\mathrm{qPlus}}$ (i.e., $A_{0}^{\mathrm{TF}}>A_{0}^{\mathrm{qPlus}}$). Moreover, the normalized capacitance, Equations (9) and (10), shows ${\bar{C}}_{0}^{\mathrm{qPlus}}=2{\bar{C}}_{0}^{\mathrm{TF}}$. These two features are consistently observed in experiments, as shown in Figure 3.

Although the dynamic characteristics vary by probe type (Equations (5)–(10)), we find two intrinsic constants independent of the probe type, defined as

(11) $$\begin{array}{cc} & U_{1}\equiv \frac{\alpha \beta R_{\mathrm{out}}}{k}=\frac{A_{m}^{\mathrm{TF}}/V_{0}}{2w_{0}^{\mathrm{TF}}Q^{\mathrm{TF}}}=\frac{A_{m}^{\mathrm{qPlus}}/V_{0}}{w_{0}^{\mathrm{qPlus}}Q^{\mathrm{qPlus}}}, \end{array}$$

(12) $$\begin{array}{cc} & U_{2}\equiv \frac{C_{0}k}{\alpha \beta }=2{\bar{C}}_{0}^{\mathrm{TF}}={\bar{C}}_{0}^{\mathrm{qPlus}}. \end{array}$$

**Figure 5:**
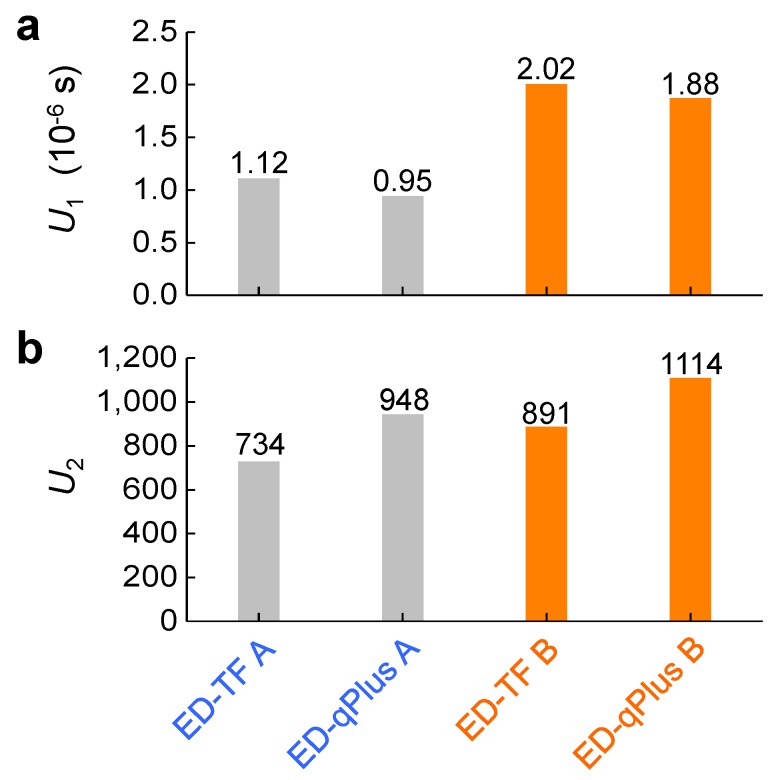
Two constants $U_{1}$ (**a**, Equation (11)) and $U_{2}$ (**b** Equation (12)) derived from the electromechanical model (Figure 4) for the electrically driven tuning fork and the qPlus sensor. Although the qPlus and its original bare tuning fork exhibit very different dynamic characteristics (Figure 3), the two constants $U_{1}$ and $U_{2}$, made of their combinations, show almost similar values for qPlus and tuning fork, as expected from our model (Figure 4).

Figure 5 shows the constants $U_{1}$ and $U_{2}$ for the TF and qPlus, which show similar values. This discrepancy could be attributed either to imperfect bonding between one prong of the qPlus and the supporting wall (Figure 1b), or to the base of the qPlus partially covered with glue. While our model is linear, the nonlinearity shown by Dagdeviren et al. [20] could be partially responsible for the discrepancy in Figure 5; the resonance frequency of oscillating probes can change by a few hertz when changing the oscillation amplitude by nearly two orders of magnitude. In addition, one could derive more accurate formulas for $U_{1}$ and $U_{2}$ (Equations (11) and (12)) to reduce the difference. Further, a new model incorporating a previously developed mechanical model [22,23,24,25] and our electromechanical model could enhance the accuracy. Although the exact values of the characteristics of the qPlus can vary depending on specifics of fabrication, the overall expected changes in the characteristics (Equations (5)–(8)) are consistent with experimental observation (Figure 3).

The present electromechanical model (Figure 4) includes the mechanical constants of the TF, that are modeled as electrical components in the traditional model (Figure 1c), and thus our model, using the mechanical motion of prongs, explains why the dynamic features such as peak amplitude and quality factor vary from TF to qPlus (Equations (5)–(8)). In addition, we modeled the quartz resonator as the coupled motion of single particles, and the model does not describe continuum mechanics and associated higher modes of the prongs’ motion [27], which could be important for nonlinear multi-mode measurements [28].

## 4. Conclusions

In summary, we presented an electromechanical model (Figure 4) that comprehensively describes the dynamic responses of the ED-TF and ED-qPlus. While the traditional equivalent circuit model reproduces the experimentally observed dynamic responses, the traditional model, employing electrical components, does not predict how the dynamic characteristics change from TF to qPlus. Based on the mechanical motion of the two prongs of the TF, our model quantitatively predicts the changes in peak amplitude, resonance frequency, quality factor, and the normalized capacitance from the TF to its qPlus configuration, and we experimentally verified these changes in the dynamic characteristics.

Our model and analysis could be helpful in using the ED-TF or ED-qPlus as a quantitative force sensor. The TF or qPlus as a force sensor requires relevant dynamic characteristics (e.g., quality factor) depending on environmental conditions. For example, one could use the ED-qPlus in amplitude-modulation mode, which could enhance imaging speed with reduced quality factor with respect to its original TF. Furthermore, one could use Equation (10) to calculate the elastic constant of the prong, which is important for accurate force measurement [29,30].

## Appendix A

From the electromechanical model (Figure 4), the equations of motion of the two prongs and the base are given by

(A1) $$\begin{array}{cc} & m_{1}{\ddot{x}}_{1}=-b{\dot{x}}_{1}-k\left(x_{1}-x_{B}\right)+F_{0}e^{iwt}, \end{array}$$

(A2) $$\begin{array}{cc} & m_{2}{\ddot{x}}_{2}=-b{\dot{x}}_{2}-k\left(x_{2}-x_{B}\right)-F_{0}e^{iwt}, \end{array}$$

(A3) $$\begin{array}{cc} & m_{B}{\ddot{x}}_{B}=-b_{B}{\dot{x}}_{B}-k_{B}x_{B}-k\left(x_{B}-x_{1}\right)-k\left(x_{B}-x_{2}\right), \end{array}$$

where the motions of prongs, $x_{1}$ and $x_{2}$, represent the deflections of the upper and lower prongs, respectively. For a bare TF, $m_{1}=m_{2}\equiv m$, the motion of two prongs exhibits the antisymmetric motion, two prongs moving in opposite direction with same speed. Thus, the resonance frequency and the quality factors are simply given by $w_{0}^{\mathrm{TF}}=w_{0}=\sqrt{k/m}$ and $Q^{\mathrm{TF}}=k/\left(bw_{0}\right)$.

In order to obtain the motion of the qPlus, we simultaneously solve Equations (A1)–(A3) and obtain the motion of the upper prong $x_{1}$ for large $m_{2}$ (i.e., $m_{2}\to \infty$). Notice that in the limit of $m_{2}\to \infty$, the motion of $x_{2}$ is inhibited so that the motion of upper prong $x_{1}$ is also obtained by assuming $x_{2}=0$ in Equations (A1)–(A3), which gives the same result, as follows:

(A4) $$\begin{array}{cc} A_{e}^{\mathrm{qPlus}}= & \mathrm{Abs}\left[\left(\frac{1}{1-\frac{\frac{k_{B}}{k}+2+\frac{b_{B}}{b\;Q^{2}}+L}{\frac{k_{B}}{k}+1}{\left(\frac{w}{w_{0}}\right)}^{2}+i\left(\frac{w}{w_{0}Q}\frac{\frac{k_{B}}{k}+H+1-\frac{m_{B}}{m_{1}}}{\frac{k_{B}}{k}+1}\right)}+\frac{C_{0}k}{\alpha \beta }\right)\frac{\alpha \beta wV_{0}R_{\mathrm{out}}}{k}\right], \end{array}$$

with

(A5) $$\begin{array}{c} L\equiv \frac{m_{B}}{m_{1}}\left(1-{\left(\frac{w}{w_{0}}\right)}^{2}\right), \end{array}$$

(A6) $$\begin{array}{c} H\equiv \frac{b_{B}}{b}\left(1-{\left(\frac{w}{w_{0}}\right)}^{2}\right). \end{array}$$

Here, the *L* and *H* vary with *w*, but they can be approximated by constants within a given bandwidth $\Delta w∼w_{0}/Q$. Then, we can estimate the dynamic characteristics of the qPlus from Equation (A4), such as

(A7) $$\begin{array}{cc} & \frac{w_{0}^{\mathrm{qPlus}}}{w_{0}^{\mathrm{TF}}}\approx \sqrt{\frac{\frac{k_{B}}{k}+1}{\frac{k_{B}}{k}+2+\frac{b_{B}}{b\;Q^{2}}+L}}\;, \end{array}$$

(A8) $$\begin{array}{cc} & \frac{Q^{\mathrm{qPlus}}}{Q^{\mathrm{TF}}}\approx \frac{\sqrt{\frac{k_{B}}{k}+1}\sqrt{\frac{k_{B}}{k}+2+\frac{b_{B}}{b\;Q^{2}}+L}}{\frac{k_{B}}{k}+H+1-\frac{m_{B}}{m}}. \end{array}$$

The geometrical dimensions of the prong and the base are not so different such that $m_{B}∼m_{1}$. In addition, we assume the base damping $b_{B}$ is much larger than the damping *b* (see Figure 4), such as $b_{B}/b\geq 10^{5}$. Also, we notice that $1-{\left(\frac{w}{w_{0}}\right)}^{2}∼2/Q$ for Q≥1000 (see Figure 3b for the TF). Under these conditions, we estimate *L* and *H*,

(A9) $$\begin{array}{cc} & \left|L\right|∼\frac{m_{B}}{m_{1}}\frac{2}{Q}\ll 1,\;\mathrm{if}\;\frac{m_{B}}{m_{1}}∼1, \end{array}$$

(A10) $$\begin{array}{cc} & \left|H\right|∼\frac{b_{B}}{b}\frac{2}{Q}>100,\;\mathrm{if}\;\frac{b_{B}}{b}\geq 10^{5}. \end{array}$$

Using Equations (A9) and (A10) and $\frac{b_{B}}{b\;Q^{2}}\ll 1$, Equations (A7) and (A8) are further approximated as

(A11) $$\begin{array}{cc} & \frac{w_{0}^{\mathrm{qPlus}}}{w_{0}^{\mathrm{TF}}}\approx \sqrt{\frac{\frac{k_{B}}{k}+1}{\frac{k_{B}}{k}+2}}\;, \end{array}$$

(A12) $$\begin{array}{cc} & \frac{Q^{\mathrm{qPlus}}}{Q^{\mathrm{TF}}}\approx \frac{\sqrt{\frac{k_{B}}{k}+1}\sqrt{\frac{k_{B}}{k}+2}}{\frac{k_{B}}{k}+H}\approx \frac{\frac{k_{B}}{k}}{\frac{k_{B}}{k}+H}, \end{array}$$

where we assumed the base elasticity $k_{B}$ is much stiffer than the prong’s elasticity *k*.

Equations (A11) and (A12) show that the resonance frequency $w_{0}^{\mathrm{qPlus}}$ and the quality factor $Q^{\mathrm{qPlus}}$ of the qPlus are determined by model parameters $k_{B}/k$ and *H*. Inversely, from Equations (A11) and (A12), one can determine the model parameters $k_{B}/k$ and *H* using the experimentally measured resonance frequency $w_{0}^{\mathrm{qPlus}}/w_{0}^{\mathrm{TF}}$ and the quality factor $Q^{\mathrm{qPlus}}/Q^{\mathrm{TF}}$ as follows:

(A13) $$\begin{array}{cc} & \frac{k_{B}}{k}\approx \frac{2{\left(\frac{w_{0}^{\mathrm{qPlus}}}{w_{0}^{\mathrm{TF}}}\right)}^{2}-1}{1-{\left(\frac{w_{0}^{\mathrm{qPlus}}}{w_{0}^{\mathrm{TF}}}\right)}^{2}}, \end{array}$$

(A14) $$\begin{array}{cc} & H\approx \frac{k_{B}}{k}\frac{Q^{\mathrm{TF}}}{Q^{\mathrm{qPlus}}}\left(1-\frac{Q^{\mathrm{qPlus}}}{Q^{\mathrm{TF}}}\right). \end{array}$$

Using experimentally measured quantities such as $w_{0}^{\mathrm{qPlus}}/\left(2\pi \right)=32,420.9\;\mathrm{Hz}$, $w_{0}^{\mathrm{TF}}/\left(2\pi \right)=32,756.5\;\mathrm{Hz}$, $Q^{\mathrm{qPlus}}=959$, and $Q^{\mathrm{TF}}=4675$ (Figure 3, ED-TF A), we obtain $k_{B}/k=47$ and H=182. Notice that the assumption in Equation (A10), H≥100, and $k_{B}/k>10$ in Equation (A12) are reproduced.

The qPlus configuration could be also obtained by a large value of *k* of the spring connecting $m_{2}$. In that condition, the mass $m_{2}$ moves along with $m_{B}$, and thus both $m_{B}$ and $m_{2}$ behave as one body with $m_{B}+m_{2}$. The coupled motions of the upper prong with mass $m_{1}$ and the effective base with $m_{B}+m_{2}$ determine the qPlus characteristics such as $w_{0}^{\mathrm{qPlus}}$ and $Q^{\mathrm{qPlus}}$. Here, the effective base mass $m_{B}+m_{2}$ is not so different from the original mass of the base $m_{B}$, since $m_{B}∼m_{2}=m_{1}$ (Equation (A9)). Note that in the case of the qPlus obtained by $m_{2}\to \infty$, the dynamic characteristics $w_{0}^{\mathrm{qPlus}}$ and $Q^{\mathrm{qPlus}}$ are determined by the coupled motions of the upper prong with mass $m_{1}$ and the base with $m_{B}$, in which the interactions made by $k_{B}$ and $b_{B}$ dominate over the interaction by the spring connecting $m_{2}$, that is, $k_{B}/k∼10$ and also $b_{B}/b∼10^{5}$, as shown earlier. Therefore, whether a qPlus is made by $m_{2}\to \infty$ or by a large value of *k* of the spring connecting $m_{2}$, the dynamic characteristics are almost the same. This feature is also predicted by Equations (A11) and (A12), where they do not include the mass information of the prong and the base.
